# Excellent option for mass testing during the SARS-CoV-2 pandemic: painless self-collection and direct RT-qPCR

**DOI:** 10.1186/s12985-021-01567-3

**Published:** 2021-05-04

**Authors:** Eva Kriegova, Regina Fillerova, Milan Raska, Jirina Manakova, Martin Dihel, Ondrej Janca, Pavel Sauer, Martina Klimkova, Petra Strakova, Petr Kvapil

**Affiliations:** 1 Department of Immunology, OLGEN, Faculty of Medicine and Dentistry, Palacky University Olomouc and University Hospital Olomouc, Hnevotinska 3, 77900 Olomouc, Czech Republic; 2 Department of Microbiology, Faculty of Medicine and Dentistry, Palacky University Olomouc and University Hospital Olomouc, Olomouc, Czech Republic; 3 Hospital Sumperk, Šumperk, Czech Republic; 4 Veterinary Research Institute, Brno, Czech Republic; 5 Institute of Applied Biotechnologies a.s., Prague, Czech Republic

**Keywords:** COVID-19, PCR diagnostics, Self-collection, Mass molecular testing, Nasal mid-turbinate swab, Post-pandemic era

## Abstract

The early identification of asymptomatic yet infectious cases is vital to curb the 2019 coronavirus (COVID-19) pandemic and to control the disease in the post-pandemic era. In this paper, we propose a fast, inexpensive and high-throughput approach using painless nasal-swab self-collection followed by direct RT-qPCR for the sensitive PCR detection of severe acute respiratory syndrome coronavirus 2 (SARS-CoV-2). This approach was validated in a large prospective cohort study of 1038 subjects, analysed simultaneously using (1) nasopharyngeal swabs obtained with the assistance of healthcare personnel and analysed by classic two-step RT-qPCR on RNA isolates and (2) nasal swabs obtained by self-collection and analysed with direct RT-qPCR. Of these subjects, 28.6% tested positive for SARS-CoV-2 using nasopharyngeal swab sampling. Our direct RT-qPCR approach for self-collected nasal swabs performed well with results similar to those of the two-step RT-qPCR on RNA isolates, achieving 0.99 positive and 0.98 negative predictive values (cycle threshold [Ct] < 37). Our research also reports on grey-zone viraemia, including samples with near-cut-off Ct values (Ct ≥ 37). In all investigated subjects (n = 20) with grey-zone viraemia, the ultra-small viral load disappeared within hours or days with no symptoms. Overall, this study underscores the importance of painless nasal-swab self-collection and direct RT-qPCR for mass testing during the SARS-CoV-2 pandemic and in the post-pandemic era.

## Introduction

Despite highly promising vaccines for the 2019 coronavirus disease (COVID-19), the key to bringing the pandemic under control worldwide and normalising all aspects of daily life in the near future is to combine vaccination with existing preventive measures and effective mass testing to detect individuals in the acute phase [25]. Therefore, cheap, easy, rapid, sensitive and high-throughput testing strategies are critical. Despite the introduction of promising rapid antigen tests, RT-qPCR protocols, which can detect severe acute respiratory syndrome coronavirus 2 (SARS-CoV-2) nucleic acid in respiratory tract specimens, remain the gold standard for COVID-19 diagnostics, mainly due to their excellent sensitivity and specificity [12, 22]. Additionally, there is an urgent need to find reliable alternatives to sample collection by healthcare personnel to expand the testing capacity and to provide easier access to testing and painless sampling [26, 29]. Many challenges remain regarding the interpretation of obtained RT-qPCR data, mainly relating to grey-zone viraemia (which includes samples with near-cut-off cycle threshold [Ct] values in RT-qPCR) and the identification of variants of concern and their influence on diagnostic settings.

The present study argues for a transition to painless self-collected nasal swabs and direct RT-qPCR to accelerate and streamline COVID-19 diagnostics during the pandemic and in the post-pandemic era. Additionally, RT-qPCR results in the grey zone are discussed, as these subjects may have an ultra-low viral load without inducing a specific immune response.

## Materials and methods

**Fig. 1:**
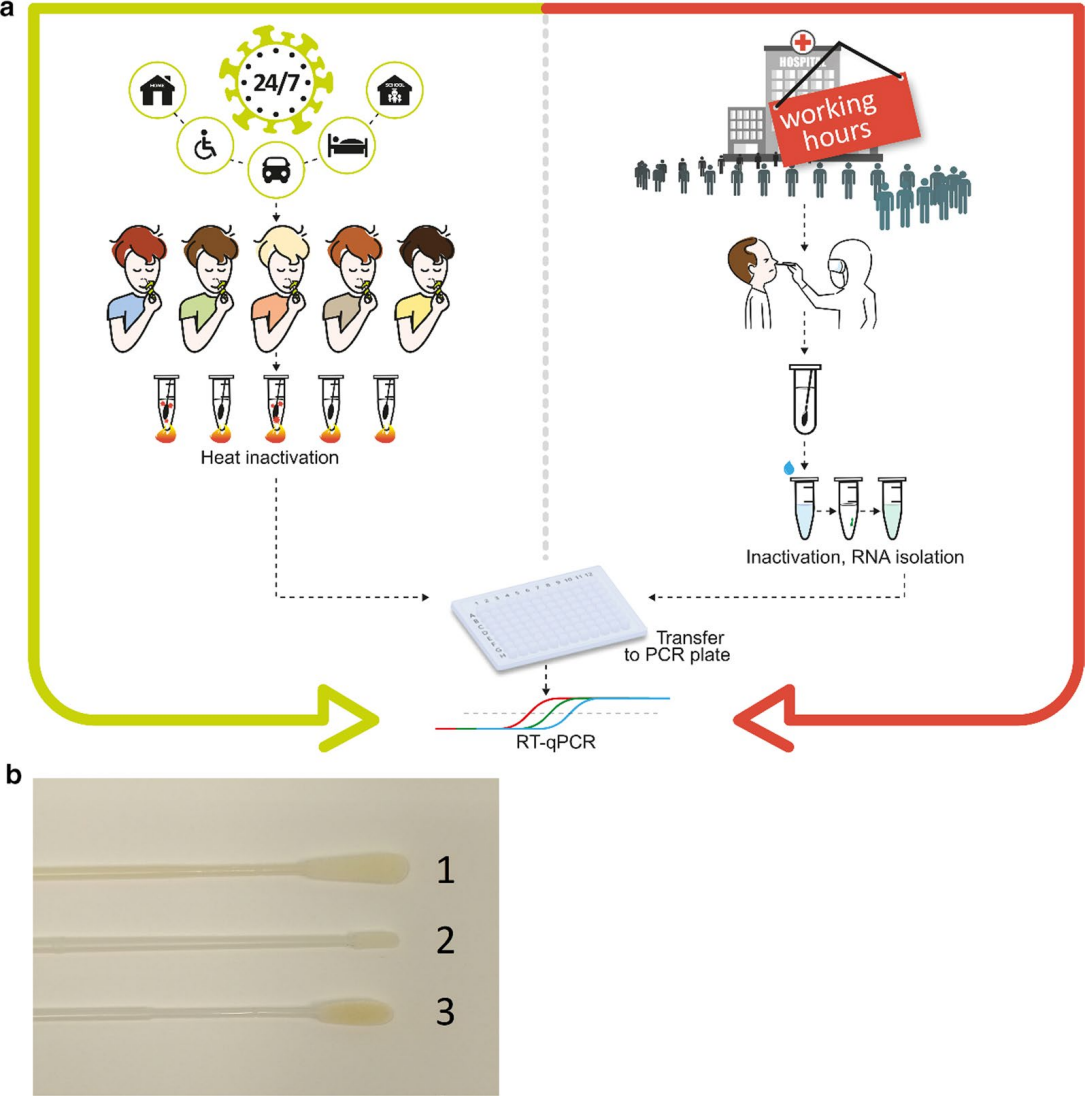
**a** Study design: comparison of nasal-swab self-collection followed by direct RT-qPCR (left panel) vs nasopharyngeal-swab  healthcare personnel-assisted sampling with two-step RT-qPCR (RNA isolation followed by PCR; right panel). **b** Nylon-flocked swab tips tested for self-collected nasal swabs. (1) FLOQSwabs MFS-98000KQ (iClean), (2) MFS-97000KQ (iClean) and 3) 520CS01 (COPAN Diagnostics Inc.)

In this prospective study performed in October and November 2020 at the testing centres of University Hospital Olomouc and Sumperk Hospital, Czechia, 1038 enrolled subjects underwent nasopharyngeal-swab sampling carried out by healthcare personnel, followed by self-collected nasal-swab sampling for SARS-CoV-2 RT-qPCR detection on the same day. All collected swabs were stored at 4 °C and analysed within 24 h of the sampling. The study design is shown in Fig. 1. The subjects signed their informed consent, approved by the Ethical Committee of University Hospital Olomouc, and completed a questionnaire comparing their comfort during both types of sampling.

Two-step RT-qPCR was performed on the nasopharyngeal swabs collected by the healthcare personnel in 2 ml of universal transport media (UTM, COPAN Diagnostics Inc.). Viral RNA isolation was performed on 200 μl of swabs in UTM using an automated nucleic acid magnetic bead extraction platform, Zybio EXM 3000 (Zybio, Shenzhen, China), and a nucleic acid extraction kit (Zybio). The final elution volume was 50 μl. RT-qPCR was then performed using a Novel Coronavirus (2019-nCoV) Real-Time Multiplex RT-PCR Kit (LifeRiver, Shanghai, China) to target the *ORF1ab, E* and *N* genes, according to the manufacturer's recommendations (20 μl Master Mix and 5 μl isolated RNA; 40 cycles) [24]. The detection limit was five copies per reaction.

For the direct RT-qPCR, nasal-swab self-collection was individually performed after instruction and under the supervision of trained personnel using nylon-flocked swab tips (FLOQSwabs MFS-98000KQ, iClean; MFS-97000KQ, iClean; 520CS01, COPAN Diagnostics Inc.) (Fig. 1). Different types of swab tip were used in this study because of the shortages caused by the COVID-19 pandemic, but all were similar in performance, as assessed by the expression of the control *RP* gene. Briefly, mid-turbinate swabbing was performed using a nylon-flocked swab tip inserted ~ 2.5-cm deep in both nasal cavities for 10 s and gently rotated. Then, the swab was immersed in 0.2 ml of COVID media in a 1.5-ml Eppendorf-type tube (part of the DIOS-RT-qPCR Kit, IABio, Czechia). Before analysis, the swab was heat inactivated at 75 °C for 10 min while being shaken, followed by spin centrifugation. Subsequently, the DIOS-RT-qPCR Kit (IABio) was used to target the *N1/N2/RP* genes (16 μl of Master Mix and 14 μl of inactivated swab eluate in COVID media; 40 cycles) [15]. The detection limit was seven copies per reaction. A comparison of the performance of the DIOS-RT-qPCR Kit, starting with the nasopharyngeal swabs leached in UTM, and classic two-step RT-qPCR on RNA isolated from the swabs had already been conducted, with the majority of samples delivering the same results in terms of positivity/negativity and Ct values in both settings [15]. To minimise the potential of false-negative results, positive (SARS-CoV-2 RNA Control 1, Twist Bioscience, USA) and negative controls (nuclease-free water) were added to each run, the *RP* gene served as an internal control for the amplification and amount of material collected with the swab in each sample. Strict laboratory procedures were established to avoid false positives, including separate laboratory rooms for the RT-qPCR setup and PCR amplification, with special shoes and coats and no transfer of disposables between the two rooms.

The presented Ct data were unnormalised for the amount of starting material. The relationship between the Ct values for both sampling methods was calculated by Pearson's rank correlation using the Analysis ToolPak add-in in Excel. The sensitivity, specificity, positive and negative predictive values and corresponding confidence intervals were calculated using a 2 × 2 table with the help of an online tool (https://www.medcalc.org/calc/diagnostic_test.php).

## Results and discussion

The COVID-19 vaccines are promising. However, worldwide vaccination will take months, and the only possible way to control the spread of COVID-19 and normalise daily life is to combine vaccination, preventive measures and effective mass testing to detect infected individuals in the acute phase. The gold standard for SARS-CoV-2 testing is still the classic two-step RT-qPCR with RNA isolation and nasopharyngeal-swab collection by healthcare personnel, as introduced at the beginning of the pandemic. In 2021, we are now facing new testing requirements: the test should be painless and easily accessible, limit the exposure of patients and staff to infection, be capable of recognising an infection with more contagious strains and be followed by fast high-throughput assays to obtain results within two hours while maintaining the desired sensitivity.

To fulfil these new requirements, we tested painless nasal-swab self-collection followed by direct one-step RT-qPCR and nasopharyngeal-swab collection and then by classic two-step RT-qPCR on RNA isolates on a cohort of 1038 subjects. Of these subjects, 297 (28.6%) were found to be positive and 741 (71.4%) negative for SARS-CoV-2 RNA using the classic two-step RT-qPCR with nasopharyngeal swabs. Upon comparing direct RT-qPCR with two-step RT-qPCR, an agreement of 94.8% (both positive and negative) was proven between the protocols. Moreover, 54 samples (5.2%) were found to be positive using only one protocol (48 samples by two-step RT-qPCR and 6 samples by direct RT-qPCR). Of the 54 positive results from only one protocol, 38 samples (70.4%) exhibited very low viral loads within the defined grey zone (Ct 37–40), corresponding to less than five SARS-CoV-2 copies per reaction, which was below the detection limits of the kits used. These results also emphasised the uneven distribution of the virus through the upper respiratory tracts (nasal, nasopharyngeal, left, right) in the case of an ultra-small viral load, in which the virus disappeared within hours or days with no symptoms. In our large real-world cohort, a specificity of 99%, sensitivity of 95%, positive predictive value of 0.99 and negative predictive value of 0.98 were achieved between the direct and two-step protocols in the samples with clear SARS-CoV-2 positivity (Ct < 37) (Table 1).

**Table 1:** The sensitivity and specificity of direct RT-qPCR on self-collected nasal swabs in samples with clear SARS-CoV-2 positivity (Ct < 37) detected by two-step RT-qPCR on nasopharyngeal swabs

	Two-step RT-qPCR on nasopharyngeal swabs		
	Positive^#^	Negative	Total
Direct RT-qPCR on nasal swab			
Positive	246	3*	249
Negative	13	776	789
Total	259	779	1038
Sensitivity	94.98% (95% CI 91.57–97.30%)		
Specificity	99.61% (95% CI 98.88–99.92%)		
Positive predictive value (PPV)	98.80% (95% CI 96.36–99.61%)		
Negative predictive value (NPV)	98.73% (95% CI 97.23–99.02%)		

^#^RT-qPCR positivity is defined as having Ct values lower than or equal to 37

^*^Direct RT-qPCR positivity for these nasal-swab samples was confirmed by two-step RT-qPCR from RNA isolates

**Fig. 2:**
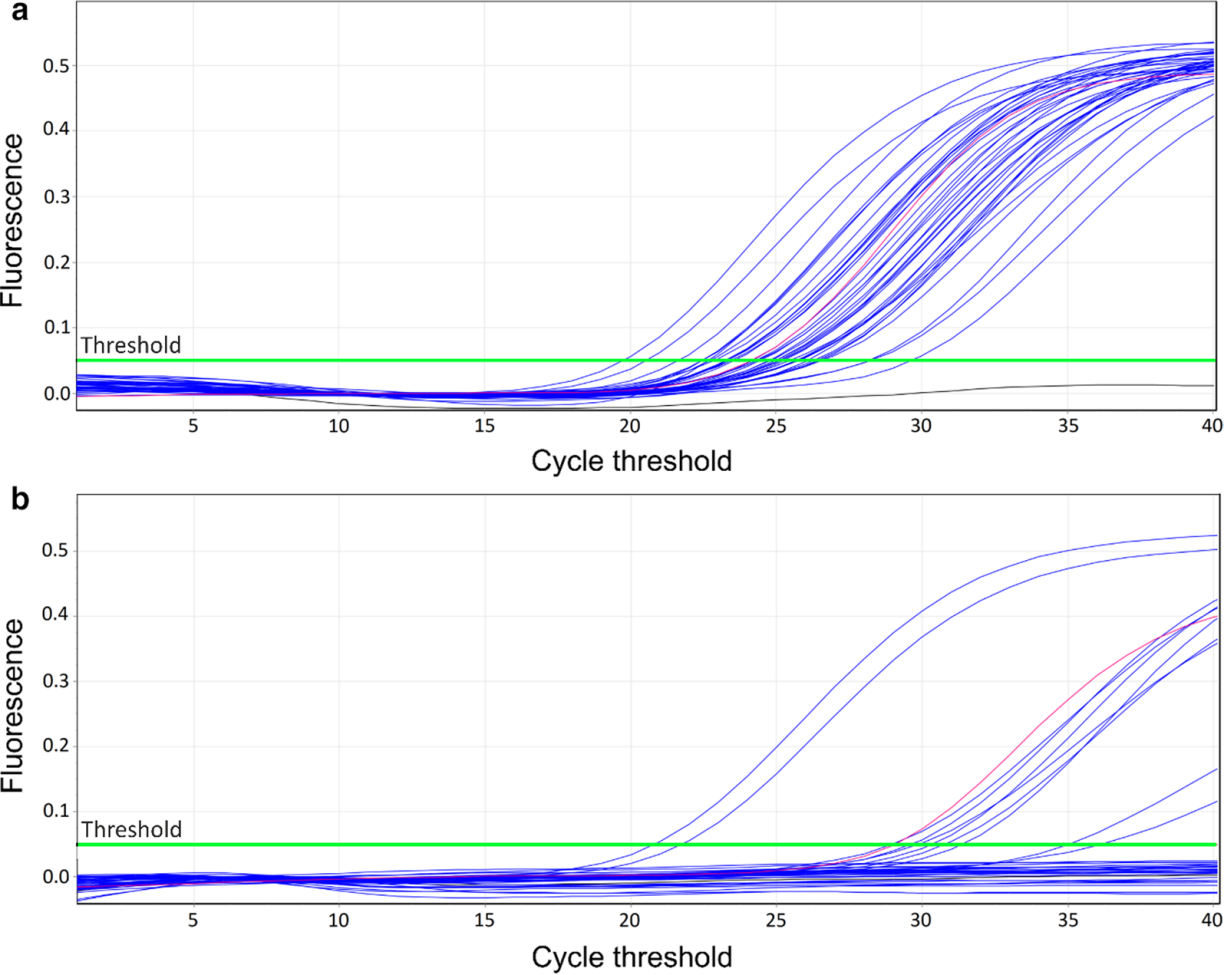
Detection of a control human RNase P (*RP*) gene (**a**) and virus-specific *N1/N2* genes* (**b**) in self-collected nasal swabs by direct RT-qPCR; the specimen was heat inactivated before the PCR analysis. To avoid false-negative results in the RT-qPCR, the human *RP* gene had to be investigated to control for proper specimen collection and amplification reaction inhibition. Positive (red line) and negative (black line) controls from direct RT-qPCR (DIOS-RT-qPCR Kit) were included in each run. *The RT-qPCR setup, primers and probe sequences have been reported previously [15]

Self-collected swabs in COVID-19 diagnostics and screenings offer significant benefits. They are easy to use and highly acceptable to the public; they limit the exposure of subjects and healthcare personnel to infection and reduce the requirement for personal protective equipment [29], as shown in the diagnostics of other respiratory pathogens [1, 14]. Regarding COVID-19, both nasopharyngeal and nasal swabs are recommended for SARS-CoV-2 RT-qPCR detection [11], and an update on 30 December 2020 added nasal mid-turbinate swabs as another acceptable method for home or on-site self-collection [5]. There is evidence that nasopharyngeal and nasal swabs have a similar performance in SARS-CoV-2 diagnostics, but nasal sampling is painless, less invasive and more comfortable [19, 20, 26, 27, 29], based on our questionnaire results, 90% of the subjects noted that the nasal swab was more comfortable, while 10% did not feel any difference between the sampling methods. Another advantage of self-collection is its independence from testing centres and the reduced COVID-19 exposure risk to healthcare personnel. It may enable sampling to be performed 24/7 on a large scale anywhere, e.g. cars, households, companies and schools (Fig. 1), and thus help identify infected subjects before sports and cultural events, festivals, parties, weddings, business meetings, etc. To avoid incorrect sampling and exclude RT-qPCR inhibition, each sample is controlled by the human *RP* control gene during direct RT-qPCR analysis, similar to the two-step RT-qPCR (Fig. 2). As shown in our real-world cohort, the majority (> 99%) of enrolled subjects obtained a sample specimen appropriate for SARS-CoV-2 analysis.

**Fig. 3:**
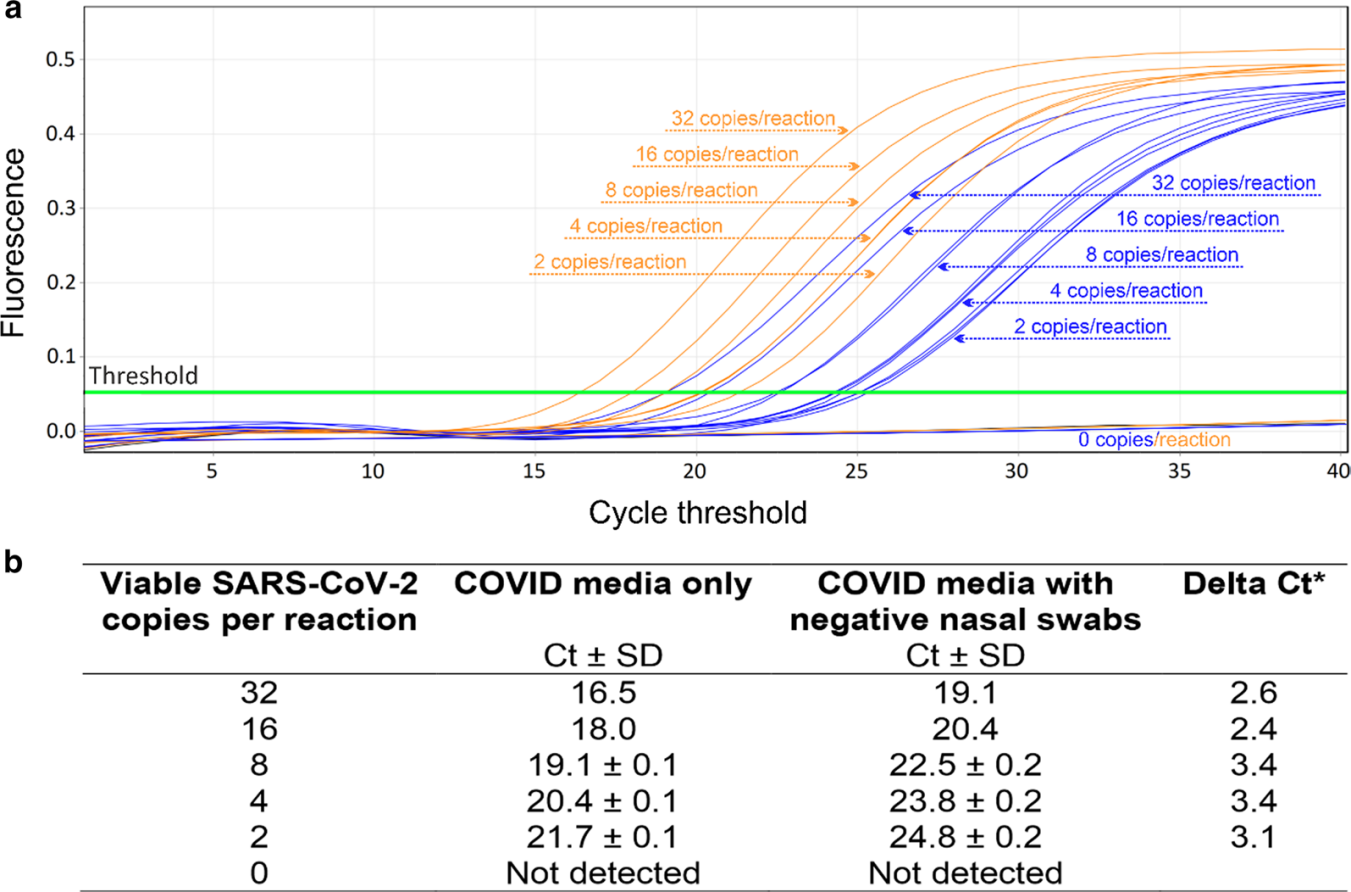
Inhibition of SARS-CoV-2 virus detection by direct RT-qPCR tested with titrated copies of viable virus culture either spiked immediately into COVID media (orange lines) or spiked into COVID media with nasal swabs from COVID-19-negative patients (blue lines). The live SARS-CoV-2 virus culture was spiked at the following concentrations: 32, 16, 8, 4, 2 and 0 copies/reaction (which translates into 1143, 571, 286, 143 and 0 copies/ml). The lowest concentrations were analysed in triplicate. The data are presented as **a** amplification curves for particular SARS-CoV-2 concentrations and **b** the corresponding Ct values. Ct: cycle threshold; SD: standard deviation; Delta Ct: the difference between the COVID medium alone and the COVID medium with negative nasal swabs for the same viral load. *A Ct difference of around 3.3 cycles corresponds to every 1log10 copies/ml change in viral load detection (2^3.3 = 9.48)

**Fig. 4:**
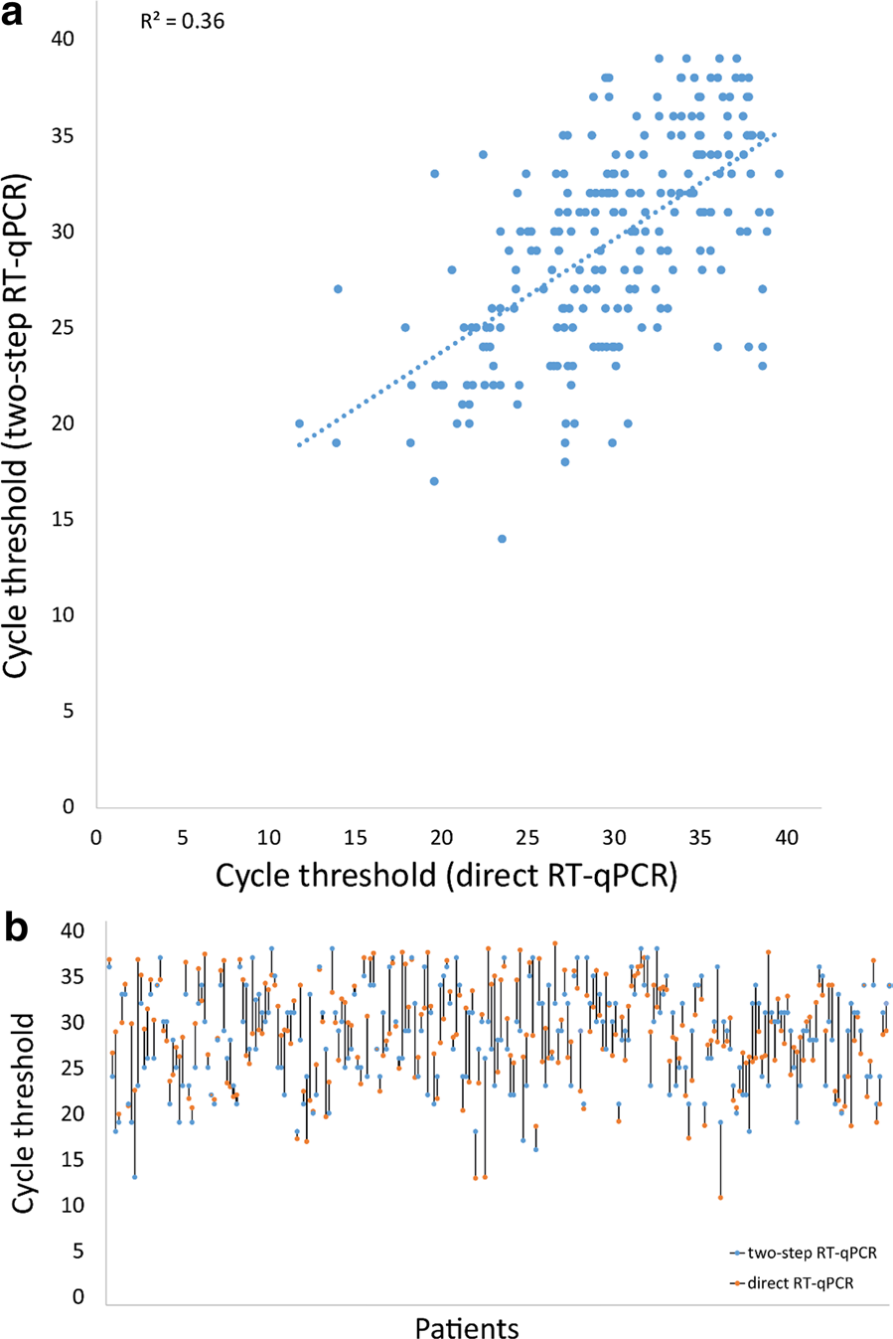
Comparison of unnormalised Ct values calculated for two-step RT-qPCR and direct RT-qPCR detection of the SARS-CoV-2 virus in all samples found to be positive by both nasopharyngeal and nasal swabs. **a** The linear regression analysis for the Ct detected for paired samples in both SARS-CoV-2 RT-qPCR methods. The x-axis shows the Ct values obtained by direct RT-qPCR, and the y-axis shows the values for the paired samples analysed by two-step RT-qPCR. **b** The distance between the Ct values from the two-step RT-qPCR (blue dot) and paired direct RT-qPCR (orange dot) represents the rate of Ct discordance between the techniques for an individual study subject

Another important step in the mass-testing setup is choosing the right analysis method. Rapid antigen testing has shown great promise for symptomatic patients; however, the sensitivity in asymptomatic and presymptomatic subjects reaches only ~ 73% [12]. Asymptomatic individuals are those who test RT-qPCR positive but experience no COVID-19 symptoms, they occur at a rate of 17–20% [3, 4, 18], with a higher prevalence in younger subjects [2]. Presymptomatic individuals are those who initially present as asymptomatic and develop symptoms days or weeks later [3, 23]. Unrecognised ‘asymptomatics’ and ‘presymptomatics’ might both contribute to a sizeable portion of the transmission events in a community because they are more likely to be a part of the community than ‘symptomatics’, who are isolated [3]. Unfortunately, we did not have the complete clinical data for our cohort of contacts and family members of patients diagnosed with COVID-19. Based on the available follow-up data in approximately a quarter of the positive individuals, we estimated that most of our positive cases were presymptomatic (~ 80%) and that approximately 20% were asymptomatic subjects (mostly younger individuals aged 18–30 years old). Therefore, mass testing in the post-pandemic era should still be based on RT-qPCR or its combination with antigen testing. For mass RT-qPCR testing, we and others have emphasised the use of direct RT-qPCR on nasopharyngeal swabs because of the minimal handling steps, speed, high throughput and simple design while maintaining the required sensitivity [13, 15, 16]. This study is the first to validate the use of self-collected nasal swabs for direct RT-qPCR in SARS-CoV-2 testing, demonstrating the required diagnostic accuracy for the detection of infected subjects. To estimate the inhibitory effect of mucosal secretions and epithelia in nasal swabs on direct RT-qPCR performance and sensitivity, we titrated viable SARS-CoV-2 viruses with a known number of copies with swabs from SARS-CoV-2-negative subjects (after thorough wiping of the nasal cavity and insertion of the swabs in COVID medium) and performed RT-qPCR (Fig. 3a). This analysis revealed a ~ tenfold inhibition of the direct RT-qPCR reaction compared to the mixing of the titrated virus with COVID medium alone, as calculated from the Ct difference reaching ~ 3 cycles for the same viral load (Fig. 3b). Nevertheless, due to the low amount of collection medium and large volume of real subject´s swabs added to the RT-qPCR reaction mixture, our approach achieved the required sensitivity requested by the FDA [11]. As shown by the scatter plots for the paired SARS-CoV-2-positive samples in Fig. 4a, nasal-swab sampling with direct RT-qPCR correlated with the nasopharyngeal samples across the whole range of Ct values. The lower correlation coefficient may be associated with the analysis of unnormalised Ct values [8] and the diversity of the distribution of the virus on different mucosal surfaces [17]. For better visualisation, Ct values for paired nasopharyngeal and nasal samples in the SARS-CoV-2-positive subjects are shown in Fig. 4b. Importantly, this direct RT-qPCR setup may also be applied to the detection of variants of concern (e.g. SARS-CoV-2 B.1.1.7, B.1.351 and P.1).

While RT-qPCR was introduced for COVID-19 diagnosis in December 2019, the interpretation of the results has not changed since then, and many laboratories report ‘positive or negative’ results based only on Ct values below 40. In general, however, there is growing evidence that diagnostic tests for ‘black or white’ decisions often do not reflect the reality of clinical settings; some values may be within the grey zone due to kit sensitivity, uncertainty about the disease status, test reliability or observer, instrumental and biological components of variance [7, 28]. In SARS-CoV-2 diagnostics, we and others have declared the diagnostic grey zone to be within the range of Ct 37–40 and have recommended the re-sampling and subsequent re-testing of samples from clinically affected sites to minimise misclassification errors [28, 30, 31]. Samples with ultra-low viral loads are often repeatedly analysed in laboratories for the final reporting of the results, which slows down the analytical process. In line with these observations, we followed 20 subjects with test results within the defined grey zone (median Ct 38.1, min–max Ct 37.0–39.9) using direct RT-qPCR on self-collected nasal swabs, all of whom became SARS-CoV-2 negative within two days in subsequent direct RT-qPCR tests (Ct > 40). Additionally, according to information given by the infected subjects, their close contacts did not become infected within the subsequent 14 days. This may suggest that the ultra-low viral loads in reported cases are effectively removed by innate immune mechanisms, thus preventing virus amplification and acute infection, with the viral antigen load below the threshold for recognition by specific immunity [6, 10]. Therefore, future research should address the association of ultra-low SARS-CoV-2 loads with infectivity, specific immunity and, particularly, the induction of neutralising antibodies. Based on the above arguments, semiquantitative results relative to viral load (high, middle and low viral load, borderline within the grey zone) instead of only qualitative RT-qPCR test results (positive × negative) would better assist clinicians in risk-stratifying patients and their contacts and choosing more appropriate quarantine conditions [9] and, eventually, more appropriate therapies [21, 32].

This study has several limitations, especially in relation to the specific conditions of COVID-19 RT-qPCR testing. Worldwide, hundreds of thousands of measurements, primarily following WHO guidelines [30, 31], are performed daily in approved diagnostic laboratories with different FDA- and CE-IVD–approved kits and/or home-based methods based on different gene sets, detection limits and instruments and using different disposables due to the shortages caused by the pandemic. Regarding the data, results are required within 48 h after sampling and are reported only qualitatively (negative/positive). The Ct values recorded internally in diagnostic laboratories are not normalised for sampling variability, and the dispersion of the data reaches up to four log units (10,000-fold). The Ct values differ for used instruments and different targets and primer/probe designs [8], which makes it difficult to perform a statistical analysis on the data obtained and compare different approaches. When comparing diagnostic kits and approaches, the only measure of quality in SARS-CoV-2 RT-qPCR diagnostics is a correct quality result in external quality control runs and the detection limit of 20 copies of SARS-CoV-2 per reaction given by the FDA [11]. In this regard, both kits used in this study met FDA requirements for the detection limit and gave the same results in terms of positivity/negativity in the external quality control runs (for a comparison of Ct values in the WHO (2020) Testing Program for the Detection of SARS-CoV-2 by PCR, see Table 2). Our study demonstrated that painless self-collection followed by direct RT-qPCR represent an excellent option for mass testing during the SARS-CoV-2 pandemic as well as for the post-pandemic era. Further enhancement of testing capacity and lowering the price per one tested subject may be achieved by the RT-qPCR pooling method followed by the re-testing of positive individual samples [33].

**Table 2:** Results of WHO EQA specimens obtained by direct RT-qPCR and classic RT-qPCR approaches during WHO (2020) EQA Testing Program for the Detection of SARS-CoV-2 by PCR in February 2021

WHO EQA specimens ID	Direct-RT-qPCR result			Two-step RT-qPCR result		
	SARS-CoV-2 result	*N1/N2* (Ct value)	SARS-CoV-2 result	*N* gene (Ct value)	*E* gene (Ct value)	*ORF1ab* (Ct value)
WHO-SC-20-01	Positive	27.7	Positive	30.5	30.1	29.2
WHO-SC-20-02	Positive	23.1	Positive	25.8	25.3	24.1
WHO-SC-20-03	Negative	ND	Negative	ND	ND	ND
WHO-SC-20-04	Negative	ND	Negative	ND	ND	ND
WHO-SC-20-05	Positive	32.3	Positive	34.0	34.6	32.5

Direct RT-qPCR was performed by heat lysed swab specimen using DIOS-RT-qPCR Kit (IABio, Czechia) and two-step RT-qPCR using Novel Coronavirus (2019-nCoV) Real-Time Multiplex RT-PCR Kit (LifeRiver, Shanghai, China) using extracted RNA from swab specimen

*ND* not detected

## Conclusion

This is the first large-scale validation study on the use of painless nasal-swab self-collection in conjunction with direct RT-qPCR, proving the diagnostic utility of this approach for mass SARS-CoV-2 testing. This fast, inexpensive and easy SARS-CoV-2 testing method could significantly increase the capacity of the test programmes needed to control the spread of COVID-19 during the pandemic and in the post-pandemic era.

## Data Availability

The data of this study are available from the corresponding author on reasonable request.

## Abbreviations

COVID-19: Coronavirus disease 2019
Ct: Cycle threshold
PCR: Polymerase chain reaction
RNA: Ribonucleic acid
RT-qPCR: Reverse transcription quantitative real-time PCR
SARS-CoV2: Severe acute respiratory syndrome coronavirus 2
SD: Standard deviation
